# QuickStats

**Published:** 2014-08-29

**Authors:** FROM THE NATIONAL CENTER FOR HEALTH STATISTICS

**Figure:**
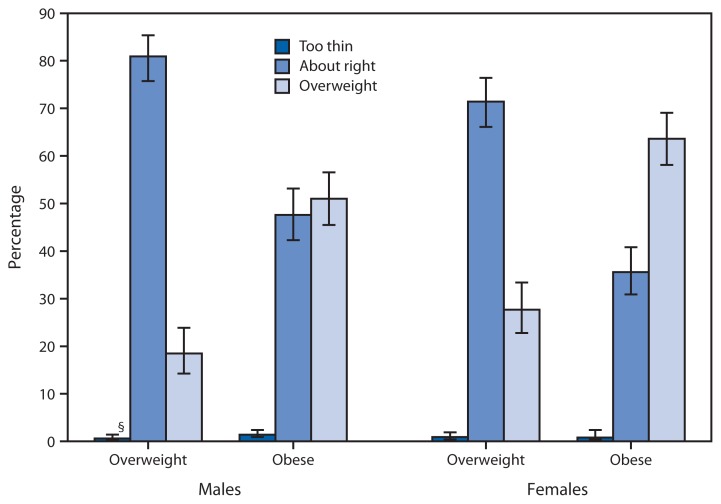
Weight Perception* Among Children and Adolescents Aged 8–15 Years, by Sex and Body Mass Index (BMI) Category^†^ — National Health and Nutrition Examination Survey (NHANES), United States, 2005–2012 * Based on responses to the question, “Do you consider yourself now to be: fat or overweight, too thin, or about the right weight?” ^†^ Overweight is defined in children and adolescents as an age-specific and sex-specific BMI ≥85th and <95th percentile of the 2000 CDC growth chart; obese is defined in children and adolescents as an age-specific and sex-specific body mass index ≥95th percentile of the 2000 CDC growth chart. ^§^ 95% confidence interval

Overweight children and adolescents aged 8–15 years were more likely to report that their weight was “about right” than report that they were “overweight,” according to NHANES data for the period 2005–2012. Among overweight females, 71.4% considered their weight “about right,” and 27.7% thought they were “overweight.” Among overweight males, 80.9% considered their weight “about right,” and 18.5% thought they were “overweight.” Among obese children and adolescents, 63.6% of females thought they were “overweight,” and 35.6% considered their weight “about right”; the percentages of obese males who thought they were “overweight” (51.0%) was roughly equal to the percentage who considered their weight “about right” (47.6%).

**Source:** Sarafrazi N, Hughes JP, Borrud L, Burt V, Paulose-Ram R. Perception of weight status in U.S. children and adolescents aged 8–15 years, 2005–2012. NCHS data brief no. 158. Hyattsville, MD: US Department of Health and Human Services, CDC; 2014. Available at http://www.cdc.gov/nchs/data/databriefs/db158.pdf.

**Reported by:** Neda Sarafrazi, PhD, vng1@cdc.gov, 301-458-4684; Steven M. Frenk, PhD.

